# Red Blood Cell Transfusion Practices for Patients With Cervical Cancer Undergoing Radiotherapy

**DOI:** 10.1001/jamanetworkopen.2021.3531

**Published:** 2021-04-05

**Authors:** Sondos Zayed, Timothy K. Nguyen, Cindy Lin, Gabriel Boldt, Sushil Beriwal, Carien L. Creutzberg, Mitchell Kamrava, Lucas C. Mendez, Vikram Velker, Corinne Doll, Amandeep Taggar, Eric Leung, David P. D’Souza

**Affiliations:** 1 Department of Radiation Oncology, London Health Sciences Centre, London, Ontario, Canada; 2 Department of Radiation Oncology, UPMC Hillman Cancer Center, Pittsburgh, Pennsylvania; 3 Department of Radiation Oncology, Leiden University Medical Centre, Leiden, the Netherlands; 4 Department of Radiation Oncology, Cedars-Sinai Medical Center, Los Angeles, California; 5 Department of Radiation Oncology, Tom Baker Cancer Centre, Calgary, Alberta, Canada; 6 Department of Radiation Oncology, Odette Cancer Centre, Sunnybrook Health Sciences Centre, Toronto, Ontario, Canada

## Abstract

**Importance:**

Packed red blood cell (PRBC) transfusions are used to treat anemia in patients with cervical cancer undergoing radiotherapy (RT) owing to concerns of hypoxia-induced radioresistance. In the absence of high-quality evidence informing transfusion practices for patients receiving external beam RT (EBRT) and brachytherapy, various arbitrary hemoglobin target levels are used worldwide.

**Objective:**

To develop consensus statements to guide PRBC transfusion practices in patients with cervical cancer receiving curative-intent RT with EBRT and brachytherapy.

**Design, Setting, and Participants:**

This international Delphi consensus study was completed between November 1, 2019, and July 31, 2020. A total of 63 international clinical experts in gynecologic radiation oncology were invited; 39 (62%) accepted and consented to participate. Consensus building was achieved using a 3-round anonymous Delphi consensus method. Participants rated their agreement or disagreement with statements using a 5-point Likert scale. An a priori threshold of 75% or more was required for consensus.

**Main Outcomes and Measures:**

The preplanned primary outcome of this study was to assess hemoglobin transfusion thresholds and targets for both EBRT and brachytherapy by expert consensus.

**Results:**

Response rates of 100% (39 of 39), 92% (36 of 39), and 97% (35 of 36) were achieved for the first, second, and third rounds of surveys, respectively. Twenty-three experts (59%) practiced in Canada, 11 (28%) in the United States, 3 (8%) in South America, 1 (3%) in Europe, and 1 (3%) in Asia. Consensus was reached for 44 of 103 statements (43%), which were combined to form the final 27-statement consensus guideline. No specific hemoglobin transfusion threshold was agreed on by consensus for EBRT or brachytherapy. By consensus (89% [31 of 35]), a hemoglobin transfusion target for patients who receive a PRBC transfusion should be 9 g/dL or more and less than 12 g/dL.

**Conclusions and Relevance:**

This study presents the first international expert consensus guideline informing PRBC transfusion practices for patients with cervical cancer undergoing EBRT and brachytherapy. A minimum hemoglobin transfusion target of 9 g/dL was endorsed to balance tumor radiosensitivity with appropriate use of a scarce resource. Randomized clinical trials are required to evaluate the optimal transfusion threshold and target that maximize clinical benefit in this patient population.

## Key Points

**Question:**

What is the hemoglobin transfusion threshold and target recommended for patients with cervical cancer undergoing curative-intent radiotherapy (RT)?

**Findings:**

In this international Delphi consensus study, 39 experts in gynecologic radiation oncology did not agree on a hemoglobin transfusion threshold, highlighting significant variability in clinical practice. For both external beam RT and brachytherapy, a hemoglobin transfusion target of 9 or more g/dL and less than 12 g/dL, respectively, was agreed on by an 89% consensus.

**Meaning:**

A liberal packed red blood cell transfusion strategy was recommended by consensus to overcome hypoxia-induced radioresistance in patients with cervical cancer receiving curative RT.

## Teaser

This international Delphi consensus study developed consensus statements to guide packed red blood cell transfusion practices for patients with cervical cancer receiving curative-intent radiotherapy with external beam radiotherapy and brachytherapy.

## Introduction

Although cervical cancers are generally considered radiosensitive, underlying tumor hypoxia may be associated with radioresistance for a subset of patients.^1,2^ A low hemoglobin level in this patient population caused by anemia of chronic disease, vaginal bleeding, and/or concurrent chemotherapy has been associated with poor local control rates, despite definitive radiotherapy (RT).^3^ Several studies have described an association between hemoglobin level and hypoxia, although the direct mechanism of the association remains obscure.^4,5,6^ Despite conflicting evidence regarding its benefit, the administration of packed red blood cell (PRBC) transfusion(s) before RT for patients with cervical cancer and anemia has historically been associated with higher local control and overall survival (OS).^7,8,9,10^ Although this practice is not informed by any recent randomized data, it is hypothesized to increase tumor radiosensitivity by improving tumor oxygenation, thereby facilitating the formation of reactive oxygen species that indirectly induce permanent DNA damage and trigger cancer cell death.^7,9,11,12,13,14,15^ This rationale is often used to justify the use of PRBC transfusions prior to and during RT for malignant neoplasms of the cervix, with the purpose of maintaining hemoglobin levels above a prespecified and often arbitrary threshold throughout treatment.^16^

Packed red blood cells are a finite resource and are not administered without risk.^17^ However, to our knowledge, no guidelines currently exist to guide PRBC transfusion practices for patients with cervical cancer undergoing RT, owing to the absence of high-quality evidence. Given that cervical cancer is highly prevalent, particularly in the developing world,^18^ the impact of guidance from experts on PRBC use in this setting is potentially substantial. The objective of this study was to develop an international consensus guideline using the Delphi method^19^ to inform PRBC transfusion practices for patients with cervical cancer receiving curative-intent RT.

## Methods

### Systematic Review

**Figure 1.**
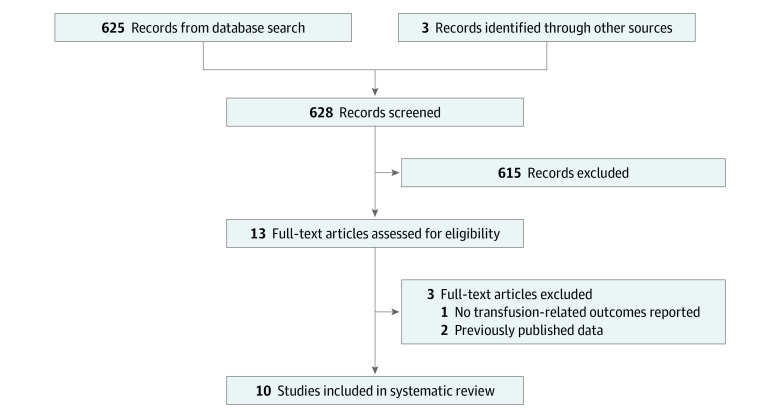
Systematic Review PRISMA Flow Diagram

This international Delphi consensus study was completed between November 1, 2019, and July 31, 2020. We performed a systematic review based on the Preferred Reporting Items for Systematic Reviews and Meta-analyses (PRISMA) reporting guideline. The results of this systematic review would allow for a comprehensive evaluation of the available evidence to inform the first Delphi survey. The PubMed (Medline), EMBASE, and Cochrane Library databases were queried from their respective dates of inception until January 2019 using search terms for RT, PRBC transfusion, and cervical cancer (eMethods 1 in the Supplement). Peer-reviewed studies in the English language reporting on patients with cervical cancer receiving RT and PRBC transfusion were included. Non–peer-reviewed correspondences, studies with 20 patients or fewer, and studies with patients receiving long-term transfusions for a nonmalignant neoplasm were excluded. Two investigators (S.Z. and C.L.) independently screened titles and abstracts and performed full-text reviews of eligible studies (Figure 1). The full texts that met all the inclusion and exclusion criteria underwent data extraction. Studies from the same institution were reviewed to assess any potential overlap, such as secondary analyses of previously reported data. The modified Oxford Centre for Evidence-Based Medicine levels of evidence criteria were used to rate the quality of evidence for every study.^20^ Study characteristics and outcomes were summarized with median values and ranges or mean (SD) values, as appropriate. This Delphi study was reviewed and approved by the Western University Health Science Research Ethics Board. Written informed consent was obtained from all participants.

### Delphi Method

Expert radiation oncologists specializing in the treatment of gynecologic malignant neoplasms in various countries were invited to form a consensus panel. Candidate selection targeted reputable opinion leaders in the management of cervical cancer, as demonstrated by publication output and/or clinical trial leadership. The final list of invited experts was agreed on by 2 authors (S.Z. and D.P.D.). The 3 investigators facilitating the Delphi process (S.Z., T.K.N., and D.P.D.) did not participate in the expert panel and did not provide opinions on any of the questions asked or on the statements that comprised the resulting guidelines.

Three iterative rounds of consensus building were completed using online surveys based on the Delphi method (eFigure in the Supplement).^19^ Study data were collected and managed using REDCap (Research Electronic Data Capture), a secure web-based software platform designed to support data capture for research studies, hosted at the London Health Sciences Centre in London, Ontario, Canada.^21,22^ Results from the systematic review were used to inform the first survey in which participants answered open-ended questions on the topics of (1) the timing of hemoglobin measurement, (2) transfusion thresholds and targets, (3) transfusion timing, and (4) follow-up. Threshold was defined as the level at or below which an intervention would be indicated (eMethods 2 in the Supplement). Target was defined as the goal that was selected as the aim of an intervention. Interstitial brachytherapy was defined as the insertion of needles using a hybrid system or a perineal template. Statements for the second and third round of online surveys were informed from the previous round(s) and were answered using a 5-point Likert scale (where 1 indicated strongly agree and 5 indicated strongly disagree). For a statement to reach consensus, a prespecified threshold of 75% or higher agreement or disagreement was used.^19^ Statements on which consensus was reached were recorded and were not included in subsequent surveys. Feedback and comments were elicited from participants for every round and incorporated into subsequent rounds as the study progressed. The third and final round consisted of statements on which consensus was not reached. After the third round, statements without consensus were excluded from the final recommendations.

**Figure 2.**
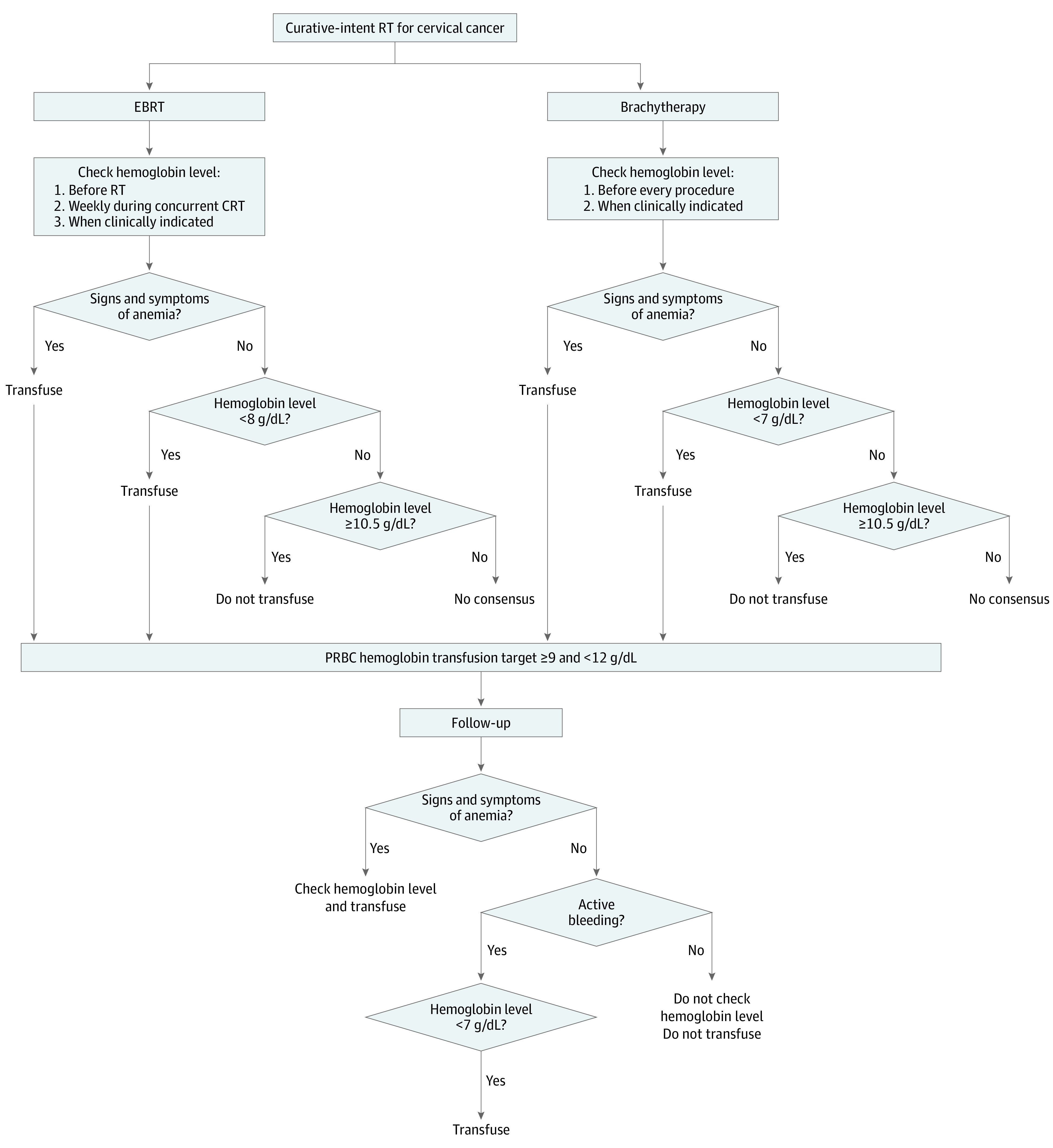
Packed Red Blood Cell (PRBC) Transfusion Algorithm for Cervical Cancer CRT indicates chemoradiotherapy; EBRT, external beam radiotherapy; and RT, radiotherapy.

All statements that reached consensus were collected and amalgamated to generate the final consensus guideline that was distributed to the expert participants for feedback in a fourth and final survey. As per the Delphi method, only minor modifications of grammar and wording were accepted at this stage, without the addition or removal of consensus statements.^19^ The final resulting guideline was used to create an algorithm outlining the endorsed decision-making process (Figure 2).

## Results

### Study Characteristics and Outcomes

Ten studies published between 1978 and 2015 met the inclusion criteria, with a total patient sample size of 5229 (range per study, 109-2454 patients).^7,8,9,10,15,16,23,24,25,26,27^ Figure 1 displays the article selection process. Eight of the 10 published articles were retrospective.^9,10,15,16,23,24,25,26^ Two were randomized clinical trials, 1 published in 1978 and 1 in 2008.^7,27^ Two articles^9,28^ reported on the same cohort of Canadian patients, and therefore only the earlier article published in 1999 by Grogan et al^9^ was included in the systematic review. The quality of evidence ratings ranged from 2 to 4 (median, 3).^20^ Articles were published on patient populations from Canada, the US, France, and South Korea. Individual study characteristics are summarized in Table 1.^7,8,9,10,15,16,23,24,25,26,27,28^

**Table 1.** Systematic Review Studies on PRBCT for Patients With Carcinoma of the Cervix

Source	Country	Study type	No.	Study population^a^	Age, mean (SD), y	Follow-up, median (range), mo	PRBCT threshold	Comparison	Results	Conclusions	Quality of evidence^**b**^
Bush et al,^7^ 1978 and Bush^8^ 1986 (reanalysis)	Canada	RCT	132	FIGO stage IIB, III cervical CA Treated with RT	NR	NR, minimum 6	Hemoglobin level <10 g/dL	Experimental group: anemia + PRBCT (n = 38); no anemia and no PRBCT (n = 28) Control group: anemia, no PRBCT (n = 25), no anemia no PRBCT (n = 41)	38 Patients received PRBCT Higher PRR in control group with anemia that did not receive PRBCT (RR, 1.85; *P* = .049) Patients without anemia in control and experimental group, and patients with anemia who received transfusion in experimental group had similar PRR (RR, 0.71, 0.84, and 0.82, respectively)	Transfusion to normal hemoglobin level may improve RT LC rate	2
Girinski et al,^23^ 1989	France	Retrospective	386	FIGO stage IIB or III cervical CA Treated with definite RT	57.7 (12.3)	NR	Hemoglobin level <8-10 g/dL before and/or during RT	NA	98 Patients received PRBCT, 80% before or during RT ≥1 hemoglobin value <10 g/dL during RT has RR = 2.0 of LR failure (*P* < .001) PRBCT during RT was an adverse prognostic factor	Anemia during RT, even if brief, is detrimental to patients PRBCT before RT or intracavitary brachytherapy may be beneficial	4
McGehee et al,^24^ 1994	United States	Retrospective	125	Invasive gynecologic CA (cervix, ovary, endometrium, or vagina) Stages I-IV Treated with surgery, RT, chemotherapy alone, or combination thereof	Transfusion: 49 (13); no transfusion: 52 (15); *P* = .26	(18-36)	Hematocrit <18% prior to RT	Patients who received PRBCT (total, n = 44; cervix only, n = 28) vs no PRBCT (total, n = 81; cervix only, n = 45)	41 Patients received RT but no PRBCT 20 Patients received RT and required PRBCT: RT alone (n = 11), surgery + RT (n = 5), CRT (n = 4) DFS poorer in women who received PRBCT (*P* < .001) Persistence or recurrence more common in PRBCT group (*P* < .001) OS inferior in women who received PRBCT (*P* = .045)	In women with gynecologic malignant neoplasm, PRBCT is associated with reduced DFS, OS, and higher rates of disease persistence or recurrence	3
Fyles et al,^15^ 2000	Canada	Retrospective	965	Cervical CA Treated with RT alone	56 (range, 21-96)	121.2 (1.2-177.6)	Hemoglobin level <12 g/dL	PRBCT given to n = 353 vs no PRBCT in n = 595	Increase in proportion of patients who received a transfusion from stage I to IVA disease Patients receiving PRBCT had 48% 5-y DFS vs 67% in patients who did not require PRBCT during RT (*P* < .001)	PRBCT does not fully counteract the adverse effect of anemia on DFS	3
Grogan et al,^9^ 1999 and Thomas et al,^28^ 2001^c^	Canada	Retrospective, multi-institutional	605	FIGO stage IB, II, III, or IVA cervical CA Treated with radical RT or CRT, ≥35 Gy	56 (range, 26-93)	41 (0-92)	Hemoglobin level <10 g/dL, <11 g/dL, or <12 g/dL (center dependent)	NA	152 Patients (25%) received ≥1 PRBCT Clinicians more likely to give PRBCT if nadir hemoglobin level <10 g/dL Stepwise increase in OS as AWNH levels increased (*P* < .001) Patients with low hemoglobin level (<12 g/dL) prior to RT that was raised to a high hemoglobin level (≥12 g/dL) with PRBCT had similar 5-y OS to patients with high hemoglobin level before RT that remained high (70% vs 74%)	Negative prognostic impact of low hemoglobin level can be overcome by PRBCT Propose maintaining hemoglobin level ≥12 g/dL during RT	4
Kapp et al,^10^ 2002	United States	Retrospective	204	FIGO stage IB-IV primary cervical CA Treated with definitive RT	Median 66 (range, 34-85)	48 (2-164)	Hemoglobin level ≤11 g/dL before and/or during RT	NA	54 Patients received PRBCT Hemoglobin level during RT was associated with DSS, PC, and MFS Patients who responded to PRBCT showed improved PC (*P* = .02) Hemoglobin level was corrected in only 18.5% of patients who received PRBCT Persistent anemia despite PRBCT showed significantly decreased DSS (*P* = .005), PC (*P* = .001), and MFS (*P* = .048)	Hemoglobin level ≤11 g/dL correction with PRBCT showed therapeutic benefit in the small subset of patients who responded to PRBCT Chronic anemia secondary to other medical illness had no effect on outcome	4
Santin et al,^16^ 2003	United States	Retrospective	130	FIGO stage IIB-III cervical CA Treated with curative-intent RT with or without chemotherapy	Transfusion: median, 51 (range, 23-88); no transfusion: median, 56 (range, 27-89); *P* = .20	Transfusion: 50; no transfusion: 49	Hemoglobin level <10.5 g/dL	PRBCT (n = 75) vs no PRBCT (n = 55)	Median OS in patients who received PRBCT of 16.1 mo vs no PRBCT of 27.9 mo (*P* = .006)	PRBCT may not improve outcomes and may be associated with poor survival	3
Lim et al,^25^ 2008	South Korea	Retrospective	119	FIGO stage IIB cervical CA Treated with RT	Median 60 (range, 23-80)	39.3 (7.6-58.4)	Hemoglobin level <10 g/dL	PRBCT (n = 32) vs no PRBCT (n = 87)	Pre-RT PRBCT had higher risk of DM (HR, 3.75; *P* = .02) and decreased OS (HR, 4.62; *P* = .03)	PRBCT was associated with poorer oncologic outcomes (OS, DM)	3
Thomas et al,^27^ 2008	Canada	RCT	109	FIGO stage IIB-IVA cervical CA and hemoglobin level <14 g/dL treated with RT alone or CRT Randomized to receive R-HUEPO (experimental group) or PRBCT (control group)	Control group: median, 50 (range, 32-78); experimental group: median, 46 (range, 25-77)	37 (9.8-50.4)	Hemoglobin level <10 g/dL in the PRBCT group, and hemoglobin level <12 g/dL in the R-HUEPO group	Experimental group: PRBCT (n = 34, 59.6%), no PRBCT (n = 23); control group: PRBCT (n = 29, 55.8%), no PRBCT (n = 23)	Ended prematurely with <25% accrual owing to concerns for increased TEs with R-HUEPO No difference in local or distant recurrence rates between groups In patients who received PRBCT, 1 (3.4%) in control group and 7 (20.6%) in R-HUEPO group had TEs (*P* = .06)	Association of hemoglobin level >12 g/dL with PFS, OS, LC in cervical CA remains undetermined	2
Bishop et al,^26^ 2015	United States	Retrospective cohort, single institution	2454	FIGO stage IA-III cervical CA treated with definitive RT or CRT	Median, 48 (range, 19-96)	61 (0-377)	NR, median minimum hemoglobin level during RT of 8.8 g/dL in patients who received PRBCT	NA	350 (14%) Patients received pre-RT PRBCT 522 (21%) Patients received PRBCT during RT PRBCT associated with poorer FFCR (*P* < .001), FFDM (*P* = .008), and DSS	Recommend transfusion if hemoglobin level <10 g/dL at diagnosis PRBCT during RT correlated with poorer outcomes	4

Abbreviations: AWNH, average weekly nadir hemoglobin level; CA, carcinoma; CRT, chemoradiotherapy; DFS, disease-free survival; DM, distant metastasis; DSS, disease-specific survival; FFCR, freedom from central recurrence; FFDM, freedom from distant metastasis; FIGO, International Federation of Gynecology and Obstetrics; HR, hazard ratio; LC, local control; LR, locoregional; MFS, metastasis-free survival; NA, not applicable; NR, not reported; OS, overall survival; PC, pelvic control; PFS, progression-free survival; PRBCT, packed red blood cell transfusion; PRR; pelvic relapse rate; RCT, randomized clinical trial; R-HUEPO, recombinant human erythropoietin; RR, relative risk; RT, radiotherapy including external beam radiotherapy and/or brachytherapy; TE, thromboembolic event.

SI conversion factors: To convert hemoglobin to grams per liter, multiply by 10.0; and hematocrit to proportion of 1.0, multiply by 0.01.

^a^ All studies were conducted before the intensity-modulated radiotherapy era and radiotherapy was therefore administered using the 4-field box technique.

^b^ Ratings of the quality of the evidence are on a scale from 1 to 5, with 5 being an opinion of respected authorities as case reports and 1 being a properly powered and conducted randomized clinical trial or systematic review with meta-analysis. This is based on the Oxford Center for Evidence-Based Medicine levels of evidence.

^c^ Thomas et al^28^ reported on the same cohort of patients previously published by Grogan et al^9^ in 1999.

Nine articles reported on patients with locally advanced cervical cancer treated with definitive RT.^7,9,10,15,16,23,25,26,27^ McGehee et al^24^ included invasive gynecologic malignant neoplasms of the cervix, ovary, endometrium, and vagina that were treated with surgery, RT, or a combination thereof. Four studies did not have a control group and therefore did not compare outcomes between patients who did and patients who did not receive a transfusion.^9,10,23,26,28^

Hemoglobin PRBC transfusion thresholds varied between studies, ranging from less than 8 g/dL to less than 12 g/dL (to convert to grams per liter, multiply by 10.0). Three studies indicated a hemoglobin level of less than 10 g/dL as their only PRBC transfusion threshold.^7,25,27^ Others used a hemoglobin level of less than 10.5 g/dL,^16^ 11 g/dL or less,^10^ or less than 12 g/dL^29^ as their transfusion threshold, whereas 2 studies documented a hemoglobin range.^9,23^ McGehee et al^24^ reported a hematocrit and Bishop et al^26^ did not report a transfusion threshold but instead described a median minimum hemoglobin level of 8.8 g/dL in patients who received transfusions. Median follow-up for the studies ranged from 6.0 to 121.2 months (median, 44.5 months). Six studies concluded that PRBC transfusion likely did not improve patient outcomes and was instead associated with poorer disease-free survival (DFS) and OS and higher rates of local recurrence as well as distant metastases.^16,23,24,25,26,29^ Conversely, 3 studies suggested that alleviating anemia using PRBC transfusions may improve local control and overcome the poor oncologic outcomes associated with a low hemoglobin level during RT.^9,10,30^ Thomas et al^27^ intended to elucidate the effect of hemoglobin levels greater than 12 g/dL on progression-free survival, OS, and local control in this patient population; however, this was not feasible owing to trial closure.

### Delphi Process

A total of 63 international experts were invited to participate in the Delphi process. Thirty-nine (62%) accepted and consented to participate. Twenty-three (59%) practiced in Canada, 11 (28%) in the United States, 3 (8%) in South America, 1 (3%) in Europe, and 1 (3%) in Asia. The median number of years of experience practicing gynecologic radiation oncology after residency was 12 years (interquartile range, 6-18 years). The median number of gynecologic consultations completed annually by each participant was 60 (interquartile range, 48-88). Most experts performed brachytherapy for cervical cancer (36 of 39 [92%]).

The response rates were 100% (39 of 39) for the first survey, 92% (36 of 39) for the second survey, and 97% (35 of 36) for the third survey. All 3 surveys were completed by 90% of the participants (35 of 39). The final consensus statements were reviewed by 32 of 36 participants (94%), who recommended grammatical, wording, and organizational edits. Of 103 statements, 44 (43%) reached consensus. These statements were amalgamated to formulate the final 27 statements included in the final consensus guideline presented in Table 2.

**Table 2.** Final International Delphi Consensus Guideline

Statement (based on linical query: a patient will be undergoing curative-intent radiotherapy treatment for cervical cancer with external beam radiotherapy and brachytherapy)	Consensus, %
External beam radiotherapy	
A. Timing of hemoglobin measurements	
1. Hemoglobin levels should be routinely checked before the start of treatment.	94
2. Hemoglobin levels should be checked weekly during treatment if the patient is receiving concurrent chemotherapy.	100
3. Hemoglobin levels should be checked when clinically indicated (eg, anemia on presentation, signs or symptoms of anemia, active bleeding, after PRBC transfusion, and decreasing hemoglobin levels)^a^	94
B. Hemoglobin transfusion threshold^b^^,^^c^^,^^d^	
1. All patients who exhibit signs and/or symptoms of anemia and/or have anemia and are actively bleeding and/or have unstable vital signs should receive a transfusion	75, 92^e^
2. EBRT should not be delayed while awaiting PRBC transfusion if the patient is asymptomatic, with stable vital signs, and hemoglobin level is ≥7 g/dL (4.34 mmol/L)	92
3. For patients with anemia who are asymptomatic, a hemoglobin level <8 g/dL (4.96 mmol/L) warrants a PRBC transfusion	100, 75^e^
4. For patients with anemia who are asymptomatic, a hemoglobin level ≥10.5 g/dL (6.52 mmol/L) does not warrant a PRBC transfusion	89, 100^e^
C. Hemoglobin transfusion target^f^	
1. Patients who receive a PRBC transfusion should have a target hemoglobin of ≥9 g/dL (5.59 mmol/L) and <12 g/dL (7.45 mmol/L)	85, 86^e^
D. Transfusion timing	
1. PRBC transfusion to the hemoglobin target level can take place before and/or during EBRT treatment if required	94, 83^e^
2. Patients should receive a transfusion at any time (before, during, or after EBRT) if they exhibit signs and/or symptoms of anemia and/or their hemoglobin level is <7 g/dL (4.34 mmol/L)	94, 100^e^
3. Patients should receive a transfusion after EBRT treatment if clinically indicated (eg, signs and symptoms of anemia present, significant unanticipated bleeding, and/or hemoglobin level <7 g/dL [4.34 mmol/L])	100
Brachytherapy	
A. Timing of hemoglobin measurements	
1. Hemoglobin levels should be routinely checked before every brachytherapy treatment	83
2. Hemoglobin levels do not need to be routinely checked after every brachytherapy treatment unless clinically indicated (eg, low hemoglobin level on presentation or decreasing hemoglobin level, significant bleeding during the brachytherapy procedure, and/or signs and/or symptoms of anemia)	89, 89, 94^e^
B. Hemoglobin transfusion threshold^g^	
1. All patients who exhibit signs and/or symptoms of anemia and/or are anemic and actively bleeding and/or have unstable vital signs should receive a transfusion	86, 92^e^
2. Patients with a significant medical history of cardiac disease (eg, myocardial infarction or congestive heart failure) should have a higher hemoglobin threshold for PRBC transfusion	75
3. Brachytherapy should not be delayed awaiting PRBC transfusion if the patient is asymptomatic, with stable vital signs, and the hemoglobin level is ≥7 g/dL (4.34 mmol/L)	83
4. For patients with anemia who are asymptomatic, a hemoglobin level <7 g/dL (4.34 mmol/L) warrants a PRBC transfusion	94, 92^e^
5. For patients with anemia who are asymptomatic, a hemoglobin level ≥10.5 g/dL (6.52 mmol/L) does not warrant a PRBC transfusion	78, 97, 78, 97^e^
C. Hemoglobin transfusion target	
1. Patients undergoing interstitial or intracavitary brachytherapy who receive a PRBC transfusion should have a target hemoglobin level of ≥9 g/dL (5.59 mmol/L) and <12 g/dL (7.45 mmol/L)	79, 89, 89^e^
2. A higher PRBC transfusion target should not be applied for patients undergoing intracavitary brachytherapy alone compared with patients receiving interstitial brachytherapy with needle insertion^h^	82
D. Transfusion timing	
1. PRBC transfusion to the hemoglobin target level should begin before brachytherapy and can continue during the procedure if the hemoglobin level reaches the prespecified transfusion threshold	78, 92, 82^e^
2. Patients should receive a transfusion after brachytherapy if clinically indicated (eg, signs and symptoms of anemia are present, active bleeding, or hemoglobin level <7 g/dL [4.34 mmol/L])	92
3. Patients should receive a transfusion at any time (before, during, or after brachytherapy treatment) if the patient has symptomatic anemia and/or their hemoglobin level is <7 g/dL (4.34 mmol/L)	97
Follow-up	
1. All patients with cervical cancer undergoing radiotherapy should be counselled on seeking medical attention as soon as possible should they develop signs or symptoms of anemia, or if they are actively bleeding	100
2. Patients who completed their radiotherapy treatment for cervical cancer do not routinely require a hemoglobin check at their first follow-up appointment	88
3. Patients who exhibit signs and/or symptoms of anemia at their first follow-up appointment require a hemoglobin check	94
4. After completing radiotherapy treatment, only patients who have anemia with symptoms, are actively bleeding, or have a hemoglobin level <7 g/dL (4.34 mmol/L) require a PRBC transfusion	81

Abbreviations: EBRT, external beam radiotherapy; PRBC, packed red blood cell.

SI conversion factor: To convert hemoglobin to grams per liter, multiply by 10.0.

^a^ Anemia for nonpregnant women: a hemoglobin concentration less than 12 g/dL, which is equivalent to 120 g/L or 7.45 mmol/L.

^b^ No consensus was reached for hemoglobin levels between 8 g/dL and less than 10.5 g/dL for EBRT.

^c^ Threshold: the level at or below which an intervention would be indicated.

^d^ Transfusion: refers to PRBC transfusion administered with the intention of improving anemia by raising the serum hemoglobin level.

^e^ More than 1 statement that reached consensus was combined into 1 statement for brevity, clarity, and comprehensiveness.

^f^ Target: the goal that was selected as the aim of an intervention.

^g^ No consensus was reached for hemoglobin levels between 7 g/dL and less than 10.5 g/dL for brachytherapy.

^h^ Interstitial brachytherapy: the insertion of needles, whether through a hybrid system or a perineal template.

### Hemoglobin Transfusion Threshold

For external beam RT (EBRT), no consensus was reached for a hemoglobin transfusion threshold between hemoglobin levels of 8 and 10 g/dL. There was, however, consensus regarding patients with asymptomatic anemia; those with a hemoglobin level less than 8 g/dL require a PRBC transfusion (75% consensus), whereas those with a hemoglobin level of 10.5 g/dL or higher do not require a PRBC transfusion (89% consensus). Similarly, for brachytherapy, no consensus was reached for a hemoglobin transfusion threshold between hemoglobin levels of 7 and 10 g/dL. For patients with asymptomatic anemia, there was consensus that a hemoglobin level less than 7 g/dL warrants a PRBC transfusion (92% consensus), whereas a hemoglobin level of 10.5 g/dL or higher did not require a PRBC transfusion (78% consensus).

### Hemoglobin Transfusion Target

For both EBRT and brachytherapy, experts reached consensus on a single range for the PRBC transfusion hemoglobin target of 9 g/dL or higher and less than 12 g/dL. A higher transfusion target should not be applied for patients undergoing interstitial brachytherapy with needle insertion (82% consensus). No consensus was reached for the timing of the first follow-up appointment after RT completion, irrespective of patient transfusion status during treatment (range, 1 week to 3 months).

## Discussion

In the absence of high-quality, contemporary evidence to inform PRBC transfusion practices for patients with cervical cancer receiving RT, to our knowledge, we conducted the first Delphi consensus study on the topic. All survey rounds of the Delphi process had very high response rates. Experts in gynecologic radiation oncology recommended PRBC transfusions for patients undergoing EBRT with a hemoglobin level less than 8 g/dL and for those receiving brachytherapy with a hemoglobin level less than 7 g/dL. Packed red bood cell transfusions were not recommended when the patient’s hemoglobin level was 10.5 g/dL or more in both cases. The lack of consensus for a distinct hemoglobin transfusion threshold for both EBRT and brachytherapy highlights significant variability in clinical practice. In the context of both EBRT and brachytherapy, the recommended transfusion target was 9 g/dL or more and less than 12 g/dL.

For hospitalized patients, a restrictive transfusion strategy is the accepted standard of care, whereby a hemoglobin transfusion threshold of 7.0 g/dL or less in asymptomatic patients is implemented.^31,32,33^ This threshold allows PRBC supplies to be conserved without compromising mortality, overall morbidity, or rate of myocardial infarctions.^31,32,33^ However, our study findings confirm that various liberal transfusion strategies (hemoglobin thresholds and targets >7.0 g/dL) continue to be used and have been recommended, by expert consensus, for patients with cervical cancer undergoing curative-intent EBRT and brachytherapy. Historically, this liberal approach to PRBC transfusion has been justified as a means to offset the negative prognostic impact of anemia, which may promote tumor hypoxia and therefore tumor radioresistance. In addition, PRBCs are often used to treat anemia caused by bone marrow suppression from concurrent chemoradiotherapy and in anticipation of further blood loss during complex brachytherapy procedures. Per the oxygen fixation hypothesis, it is also believed that maximizing tumor oxygenation may lead to the formation of more DNA-damaging oxygen-free radicals in response to ionizing radiotherapy and that, in the presence of oxygen, a peroxy radical is formed that renders such DNA damage irreparable, thereby enhancing tumor cell killing.^34^

Our systematic review highlights the paucity of evidence addressing the role of PRBC transfusions in patients with cervical cancer undergoing RT. Some studies suggested that PRBC transfusions prior to or during RT may be beneficial to offset acute anemia, thereby optimizing tumor radiosensitivity and the effectiveness of treatment.^9,10,23^ Others concluded that PRBC transfusions prior to or during RT are either not associated with improved treatment outcomes and constitute an unnecessary use of a scarce resource, or are associated with poor progression-free survival and OS.^16,24,25,28^

The first randomized clinical trial to suggest that patients with anemia and cervical cancer may benefit from PRBC transfusions dates back to 1978. With a very heterogenous patient population and a small sample size (only 38 patients received PRBC transfusion), its generalizability is limited. Another randomized clinical trial designed to evaluate the superiority of recombinant human erythropoietin to PRBC transfusion in treating anemia in patients with cervical cancer was halted prematurely owing to concerns regarding increased thromboembolic events with recombinant human erythropoietin. The impact of hemoglobin levels greater than 12.0 g/dL in patients with cervical cancer receiving RT as a secondary end point could therefore not be assessed. To our knowledge, no recent randomized clinical trial has compared restrictive and liberal PRBC transfusion strategies in this patient population.

In a study of 88 patients with cervical cancer treated with RT or chemoradiotherapy who underwent dynamic contrast-enhanced magnetic resonance imaging to quantify tumor perfusion, Mayr et al^35^ showed that the highest 5-year local recurrence rate and the lowest DFS rates were observed for patients with both low tumor perfusion, detected on dynamic contrast-enhanced magnetic resonance imaging scans, and low hemoglobin levels, defined as less than 11.2 g/dL. This finding suggests that cervical cancer radiosensitivity likely depends on both tumor perfusion and hemoglobin level before RT and that hemoglobin levels are likely directly associated with tumor perfusion.^36^ High hemoglobin levels and high tumor perfusion, which may potentially be achieved with the endorsed liberal PRBC transfusion approach, was associated with longer DFS.^35^

The DAHANCA (Danish Head and Neck Cancer Group) 5 and 7 trials randomized 465 patients with head and neck cancer and a low hemoglobin level (defined as <13 g/dL in women and <14.5 g/dL in men) to receive a PRBC transfusion prior to and during RT, or nothing.^37^ Receipt of a PRBC transfusion did not appear to improve the local recurrence rate or DFS or OS rates, implying that a low hemoglobin level was associated with worse outcomes, irrespective of transfusion status. Patients who received a transfusion were found to have worse overall DFS and OS compared with patients who did not receive a transfusion, likely owing to comorbid conditions. These findings also suggest a more complex association between hemoglobin level and hypoxia-induced radioresistance. Unlike head and neck cancers, however, cervical cancer commonly presents with abnormal vaginal bleeding from a friable tumor^38^ that further exacerbates tumor-associated and treatment-induced anemia.^39,40^ The results of the DAHANCA 5 and 7 trials, although conclusive in the context of head and neck cancer, cannot therefore be extrapolated to cervical cancer. The liberal transfusion approach supported by the consensus guidelines may at least counteract the effect of bleeding and chemotherapy-related anemia to alleviate hypoxia-induced radioresistance.

In a study examining the association between pretreatment hemoglobin level and tumor oxygenation in multiple tumors, including cervical cancer, the maximum Po_2_ and lowest hypoxic fraction values were observed in squamous cell carcinomas at a hemoglobin level between 12 and 14 g/dL in women. Any increase above this value increased the blood’s viscosity, impairing its capacity to transport oxygen and reducing tumor oxygenation.^6^ By consensus, the maximum hemoglobin transfusion target recommended was 12 g/dL, which allows for optimal oxygenation and aligns with the findings of this study. This recommendation also appropriately cautions against higher transfusion targets, which may negatively impact patient outcomes.

### Limitations

Several limitations of the systematic review warrant mention. A meta-analysis of the included studies was not feasible owing to significant heterogeneity in patient characteristics, hemoglobin thresholds for transfusion, and reported outcomes, which prevented direct pooling of results. Importantly, most of these studies were retrospective and susceptible to recall, selection, and information biases. Most were also conducted and published before radiotherapy technology evolved to adopt intensity-modulated radiotherapy as the new standard of care for modern radiotherapy treatments.^41^ Advances in the delivery of brachytherapy, such as computed tomography–based and magnetic resonance imaging–based planning techniques, have also become standard.^42^ The relative ease of dose escalation to the primary tumor without compromising the organs at risk using these methods may reduce the association of hypoxia with tumor cell radioresistance.^43,44^ It is unclear whether the currently accepted and widely practiced liberal approach to PRBC transfusions is therefore necessary as a method of improving tumor oxygenation and tumor radiosensitivity.

Several limitations of the Delphi process also warrant mention. First, no hematologists were included in the study to provide a more balanced perspective on the advantages associated with restrictive transfusion practices that are considered standard in medicine and surgery. However, hematologists often lack the knowledge and training in radiobiology required to justify the rationale for transfusion in this context. The nature of brachytherapy procedures is moreover often obscure to hematologists. These factors limit their ability to render judgements on the administration of PRBC transfusions, specifically for patients with cervical cancer. Second, to our knowledge, high-quality, generalizable level 1 evidence is not available to help inform or guide consensus. Third, a high burden of cervical cancer is found in Africa, Asia, and South America, yet substantial challenges were met with engaging experts in these regions; only 3 from South America and 1 from Asia consented to participate. Therefore, data on PRBC transfusion practices in Africa were not captured. Participation of experts in less-wealthy countries in which PRBCs are a particularly scarce resource would have contributed substantially to the results of this study. Future studies should aim to include experts from Asia, Africa, and South America for a more comprehensive depiction of PRBC transfusion practices globally. Last, the anonymous nature of the Delphi process precludes discussion and debate among participants, which may have further facilitated the consensus-building process for questions that currently remain unanswered.

## Conclusions

We present, to our knowledge, the first international expert consensus guideline informing PRBC transfusion practices for patients with cervical cancer receiving EBRT and brachytherapy. Although a hemoglobin level between at least 9 g/dL and less than 12 g/dL was endorsed as the consensus transfusion target, significant variability in clinical practice persists owing to the lack of high-level evidence. Randomized clinical trials are required to evaluate the optimal hemoglobin transfusion threshold and target that optimize oncologic outcomes while ensuring the judicious use of PRBCs.

## Notes

Supplement. **eMethods 1. **Literature Search Strategy **eFigure.** The Delphi Process **eMethods 2.** Delphi Survey Questions
