# Supplementary material for: Red Blood Cell Transfusion Practices for Patients With Cervical Cancer Undergoing Radiotherapy
Source: JAMA Netw Open. 2021 Apr 5;4(4):e213531. doi: 10.1001/jamanetworkopen.2021.3531 (PMC8022218; doi:10.1001/jamanetworkopen.2021.3531)
Supplement: Supplement. — eMethods 1. Literature Search Strategy eFigure. The Delphi Process eMethods 2. Delphi Survey Questions [file jamanetwopen-e213531-s001.pdf]

Supplementary Online Content

Zayed S, Nguyen TK, Lin C, et al. Red blood cell transfusion practices for patients with cervical cancer undergoing radiotherapy. *JAMA Netw Open.* 2021;4(4):e213531. doi:10.1001/jamanetworkopen.2021.3531

eMethods 1. Literature Search Strategy

eFigure. The Delphi Process

eMethods 2. Delphi Survey Questions

This supplementary material has been provided by the authors to give readers additional information about their work.

© 2021 Zayed S et al. *JAMA Network Open.*

eMethods 1. Literature Search Strategy

PubMed (394 results):

(hgb[tw] OR hemotherapy[tw] OR red blood cell*[tw] OR Eryhem[tw] OR hemoglobin*[tw] OR haemoglobin*[tw] OR hemoglobins[mh] OR blood transfusion[mh] OR transfusion*[tw] OR hematocrit[tw])
AND
(radiotherapy[mh] OR irradiation[tw] OR radiotherapy[tw] OR radiation[tw])
AND
(Uterine Cervical Neoplasms[mh] OR cervix[tw] OR cervical[tw])

Limits: Humans

EMBASE (230 results) & Cochrane (1 result):

(exp hemoglobin/ or hgb.mp. or hemotherapy.mp. or exp blood transfusion/ or red blood cell*.mp. or Eryhem.mp. or hemoglobin*.mp. or haemoglobin*.mp. or transfusion*.mp. or hematocrit.mp. or exp hematocrit/)

and

(radiotherapy.mp. or exp radiotherapy/ or irradiation.mp. or exp irradiation/ or exp radiation/ or radiation.mp.)

and

(exp uterine cervix tumor/ or cervix.mp. or exp uterine cervix/ orcervical.mp.)

Limit to (human and cochrane library and exclude medline journals)

© 2021 Zayed S et al. *JAMA Network Open.*

eFigure. The Delphi Process

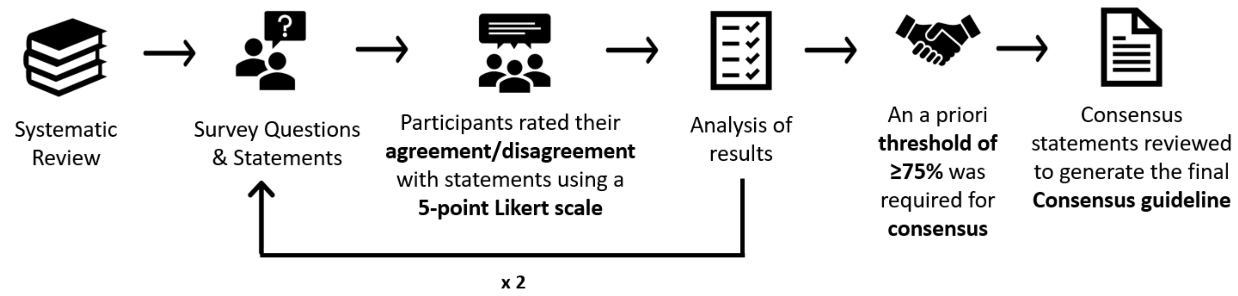

© 2021 Zayed S et al. *JAMA Network Open.*

eMethods 2. Delphi Survey Questions

SURVEY 1

- Indicate your location of practice:
- State number of years of experience treating cervical cancer as a certified Radiation Oncologist in independent practice.
- State average number of consults for gynecological malignancies you complete annually as a certified Radiation Oncologist in independent practice.
- Do you routinely preform brachytherapy for cervical cancer?
- Specify when you routinely check hemoglobin levels for EBRT: (choice=Before EBRT)
- Specify when you routinely check hemoglobin levels for EBRT: (choice=During EBRT)
- Specify when you routinely check hemoglobin levels for EBRT: (choice=After EBRT)
- Specify when you routinely check hemoglobin levels for EBRT: (choice=Never)
- For EBRT, please comment on timing, frequency of blood work and any other relevant details.
- Specify when you routinely check hemoglobin levels for brachytherapy: (choice=Before brachytherapy)
- Specify when you routinely check hemoglobin levels for brachytherapy: (choice=During brachytherapy)
- Specify when you routinely check hemoglobin levels for brachytherapy: (choice=After brachytherapy)
- Specify when you routinely check hemoglobin levels for brachytherapy: (choice=Never)
- For brachytherapy, please comment on timing, frequency of blood work and any other relevant details.
- Do you transfuse patients undergoing EBRT?
- Do you transfuse patients undergoing brachytherapy?
- What are your criteria for transfusion in patients undergoing EBRT? Please provide details and provide references where possible.
- What are your criteria for transfusion in patients undergoing brachytherapy treatment? Please provide details and provide references where possible.
- Do your criteria for transfusion for patients undergoing EBRT vs brachytherapy treatment differ? Describe why or why not.
- What is the target hemoglobin level you are aiming to reach with transfusion? Please elaborate on why this is your target.
- Does the target hemoglobin level you are aiming to reach differ between EBRT and brachytherapy?
- Please explain the rationale for why your target hemoglobin level differs between EBRT and brachytherapy.
- Please specify when you transfuse patients. (choice=Before EBRT)
- Please specify when you transfuse patients. (choice=During EBRT)
- Please specify when you transfuse patients. (choice=After EBRT)
- Please specify when you transfuse patients. (choice=Never for EBRT)
- Please specify when you transfuse patients. (choice=Before brachytherapy)
- Please specify when you transfuse patients. (choice=During brachytherapy)
- Please specify when you transfuse patients. (choice=After brachytherapy)
- Please specify when you transfuse patients. (choice=Never for brachytherapy)
- Please explain your rationale for your choice of timing for transfusion.
- Does your institution have transfusion guidelines that pertain to patients undergoing radiotherapy?
- Please summarize the transfusion guidelines provided by your institution for patients undergoing radiotherapy and provide references when possible.
- Comments:
- Complete? Yes/No

© 2021 Zayed S et al. *JAMA Network Open.*

SURVEY 2

Clinical Query: A patient will be undergoing curative intent radiation treatment for cervical cancer with External Beam Radiotherapy (EBRT) and brachytherapy.

Please consider the following definitions prior to proceeding:

- *Anemia for non-pregnant women: a hemoglobin concentration less than 12 g/dL which is equivalent to 120 g/L or 7.45 mmol/L.*
- *Threshold: The level at or below which an intervention would be indicated.*
- *Target: The goal level which was selected as the aim of an intervention.*

Please respond with your level of agreement to the following statements.

The Likert scale below will be used for every question:

Strongly Disagree	Disagree	Undecided	Agree	Strongly Agree

1, Strongly Disagree

2, Disagree

3, Undecided

4, Agree

5, Strongly Agree

EXTERNAL BEAM RADIOTHERAPY (EBRT)

EBRT: Timing of Hemoglobin Checks

- Hemoglobin levels should be routinely checked BEFORE the start of treatment.
- Hemoglobin levels should be routinely checked WEEKLY during treatment ONLY if the patient is receiving concurrent chemotherapy.
- Hemoglobin levels should be routinely checked WEEKLY during treatment even if the patient is NOT receiving concurrent chemotherapy.
- Hemoglobin levels should be checked DURING treatment ONLY if clinically indicated e.g. low hemoglobin upon presentation or dropping hemoglobin levels, concurrent chemotherapy, significant bleeding, fatigue, pallor, chest pain, etc.
- Comments

EBRT: Hemoglobin Transfusion Threshold

- All patients who exhibit signs or symptoms of anemia should be transfused.
- All patients who are anemic and actively bleeding and/or have unstable vital signs should be transfused.
- EBRT should NOT be delayed while awaiting transfusion if the patient is asymptomatic, with stable vital signs, and hemoglobin level is 7 g/dL (4.34 mmol/L) or above.
- For patients who are asymptomatically anemic, a hemoglobin level below 7 g/dL (4.34 mmol/L) warrants a blood transfusion.
- For patients who are asymptomatically anemic, a hemoglobin level below 8 g/dL (4.96 mmol/L) warrants a blood transfusion.

© 2021 Zayed S et al. *JAMA Network Open.*

- For patients who are asymptomatically anemic, a hemoglobin level below 9 g/dL (5.59 mmol/L) warrants a blood transfusion.
- For patients who are asymptomatically anemic, a hemoglobin level below 10 g/dL (6.21 mmol/L) warrants a blood transfusion.
- For patients who are asymptomatically anemic, a hemoglobin level below 10.5 g/dL (6.52 mmol/L) warrants a blood transfusion.
- For patients who are asymptomatically anemic, a hemoglobin level below 12 g/dL (7.45 mmol/L) warrants a blood transfusion.
- For patients who are asymptomatically anemic, after EBRT treatment completion, ONLY a hemoglobin level below 7 g/dL (4.34 mmol/L) warrants a blood transfusion.
- Patients with a significant past medical history for cardiac disease (e.g. MI, CHF, etc.) should have a higher hemoglobin threshold for transfusion.
- Comments

EBRT: Hemoglobin Transfusion Target

- Patients who receive a blood transfusion should be transfused to a target hemoglobin of 7 g/dL (4.34 mmol/L) or greater.
- Patients who receive a blood transfusion should be transfused to a target hemoglobin of 8 g/dL (4.96 mmol/L) or greater.
- Patients who receive a blood transfusion should be transfused to a target hemoglobin of 9 g/dL (5.59 mmol/L) or greater.
- Patients who receive a blood transfusion should be transfused to a target hemoglobin of 10 g/dL (6.21 mmol/L) or greater.
- Patients who receive a blood transfusion should be transfused to a target hemoglobin of 10.5 g/dL (6.52 mmol/L) or greater.
- Patients who receive a blood transfusion should be transfused to a target hemoglobin of 12 g/dL (7.45 mmol/L) or greater.
- For patients with a significant past medical history for cardiac disease (e.g. MI, CHF, etc.) a higher transfusion target should be used.
- Comments

EBRT: Transfusion Timing

- Transfusion to the hemoglobin target should take place BEFORE EBRT treatment.
- Transfusion to the hemoglobin target should take place BEFORE and/or DURING EBRT treatment if required.
- Transfusion to the hemoglobin target should take place ONLY DURING EBRT treatment.
- Transfusion to the hemoglobin target should take place AFTER EBRT treatment.
- Patients should be transfused AFTER EBRT treatment ONLY if clinically indicated e.g. signs and symptoms of anemia present, significant unanticipated bleeding during treatment, or hemoglobin level lower than 7 g/dL (4.34 mmol/L) etc.
- Patients should be transfused at any time (before, during, or after EBRT treatment) if the hemoglobin level is lower than 7 g/dL (4.34 mmol/L).
- Patients should be transfused at any time (before, during, or after EBRT treatment) if the hemoglobin level reaches the pre-specified hemoglobin threshold for transfusion.
- Comments

BRACHYTHERAPY

Brachytherapy: Timing of Hemoglobin Checks

- Hemoglobin levels should be routinely checked BEFORE every brachytherapy treatment.

© 2021 Zayed S et al. *JAMA Network Open.*

- Hemoglobin levels should be routinely checked within 24 hours BEFORE the start of every brachytherapy treatment.
- Hemoglobin levels should be routinely checked within 48 hours BEFORE the start of every brachytherapy treatment.
- Hemoglobin levels should be routinely checked within 72 hours BEFORE the start of every brachytherapy treatment.
- Hemoglobin levels should be routinely checked within 1 week BEFORE the start of every brachytherapy treatment.
- Hemoglobin levels do NOT need to be routinely checked DURING every brachytherapy treatment.
- Hemoglobin levels should be checked DAILY if interstitial brachytherapy applicator is in situ for more than 24 hours.
- Hemoglobin levels should be checked DAILY for Pulsed Dose Rate (PDR) brachytherapy.
- Hemoglobin levels do NOT need to be routinely checked AFTER every brachytherapy treatment.
- Hemoglobin levels should be consistently checked AFTER every interstitial brachytherapy treatment.
- Hemoglobin levels do NOT need to be routinely checked AFTER every intracavitary brachytherapy treatment.
- Hemoglobin levels should be checked AFTER a brachytherapy treatment ONLY when clinically indicated e.g. low hemoglobin upon presentation or dropping hemoglobin levels, significant bleeding, signs and symptoms of anemia, etc.
- Comments

Brachytherapy: Hemoglobin Transfusion Threshold

- All patients who exhibit signs or symptoms of anemia should be transfused.
- All patients who are anemic and actively bleeding and/or have unstable vital signs should be transfused.
- Patients with a significant past medical history for cardiac disease (e.g. MI, CHF, etc.) should have a higher hemoglobin threshold for transfusion.
- Brachytherapy should NOT be delayed awaiting transfusion if the patient is asymptomatic, stable vital signs, and hemoglobin level is 7 g/dL (4.34 mmol/L) or above.
- For patients undergoing interstitial brachytherapy with needle insertion, a higher hemoglobin threshold for transfusion should be used.

Interstitial Brachytherapy:

- For patients who are asymptomatically anemic, a hemoglobin level below 7 g/dL (4.34 mmol/L) warrants a blood transfusion.
- For patients who are asymptomatically anemic, a hemoglobin level below 8 g/dL (4.96 mmol/L) warrants a blood transfusion.
- For patients who are asymptomatically anemic, a hemoglobin level below 9 g/dL (5.59 mmol/L) warrants a blood transfusion.
- For patients who are asymptomatically anemic, a hemoglobin level below 10 g/dL (6.21 mmol/L) warrants a blood transfusion.
- For patients who are asymptomatically anemic, a hemoglobin level below 10.5 g/dL (6.52 mmol/L) warrants a blood transfusion.
- For patients who are asymptomatically anemic, a hemoglobin level below 12 g/dL (7.45 mmol/L) warrants a blood transfusion.

Intracavitary Brachytherapy

- For patients who are asymptomatically anemic, a hemoglobin level below 7 g/dL (4.34 mmol/L) warrants a blood transfusion.

© 2021 Zayed S et al. *JAMA Network Open.*

- For patients who are asymptomatically anemic, a hemoglobin level below 8 g/dL (4.96 mmol/L) warrants a blood transfusion.
- For patients who are asymptomatically anemic, a hemoglobin level below 9 g/dL (5.59 mmol/L) warrants a blood transfusion.
- For patients who are asymptomatically anemic, a hemoglobin level below 10 g/dL (6.21 mmol/L) warrants a blood transfusion.
- For patients who are asymptomatically anemic, a hemoglobin level below 10.5 g/dL (6.52 mmol/L) warrants a blood transfusion.
- For patients who are asymptomatically anemic, a hemoglobin level below 12 g/dL (7.45 mmol/L) warrants a blood transfusion.
- Comments

Brachytherapy: Hemoglobin Transfusion Target

- For patients with a significant past medical history for cardiac disease (e.g. MI, CHF, etc.) a higher transfusion target should be used.
- A higher transfusion target should be applied for patients undergoing interstitial brachytherapy with needle insertion compared to intracavitary brachytherapy alone.
- Patients undergoing Pulsed Dose Rate (PDR) brachytherapy should maintain a hemoglobin level of 10 g/dL (6.21 mmol/L) or greater throughout treatment.

Interstitial Brachytherapy:

- Patients who receive a blood transfusion should be transfused to a target hemoglobin of 7 g/dL (4.34 mmol/L) or greater.
- Patients who receive a blood transfusion should be transfused to a target hemoglobin of 8 g/dL (4.96 mmol/L) or greater.
- Patients who receive a blood transfusion should be transfused to a target hemoglobin of 9 g/dL (5.59 mmol/L) or greater.
- Patients who receive a blood transfusion should be transfused to a target hemoglobin of 10 g/dL (6.21 mmol/L) or greater.
- Patients who receive a blood transfusion should be transfused to a target hemoglobin of 10.5 g/dL (6.52 mmol/L) or greater.
- Patients who receive a blood transfusion should be transfused to a target hemoglobin of 12 g/dL (7.45 mmol/L) or greater.

Intracavitary Brachytherapy

- Patients who receive a blood transfusion should be transfused to a target hemoglobin of 7 g/dL (4.34 mmol/L) or greater.
- Patients who receive a blood transfusion should be transfused to a target hemoglobin of 8 g/dL (4.96 mmol/L) or greater.
- Patients who receive a blood transfusion should be transfused to a target hemoglobin of 9 g/dL (5.59 mmol/L) or greater.
- Patients who receive a blood transfusion should be transfused to a target hemoglobin of 10 g/dL (6.21 mmol/L) or greater.
- Patients who receive a blood transfusion should be transfused to a target hemoglobin of 10.5 g/dL (6.52 mmol/L) or greater.
- Patients who receive a blood transfusion should be transfused to a target hemoglobin of 12 g/dL (7.45 mmol/L) or greater.
- Comments

Brachytherapy: Transfusion Timing

© 2021 Zayed S et al. *JAMA Network Open.*

- Transfusion to the hemoglobin target should take place BEFORE each brachytherapy treatment.
- Transfusion to the hemoglobin target should take place BEFORE and DURING the brachytherapy treatment if required.
- Transfusion to the hemoglobin target should take place ONLY DURING the brachytherapy treatment.
- Patients should be transfused AFTER brachytherapy if clinically indicated e.g. signs and symptoms of anemia present, active bleeding, or hemoglobin level less than 7 g/dL (4.34 mmol/L).
- Patients should be transfused at any time (before, during, or after brachytherapy treatment) if the hemoglobin level reaches the pre-specified transfusion threshold.
- Comments

FOLLOW-UP

- Indicate when you generally schedule the FIRST follow-up for patients who completed their radiation treatment for cervix cancer:
- Indicate when you would follow-up on patients who received at least one blood transfusion throughout their radiation treatment for cervix cancer:
- Patients who received at least one blood transfusion throughout their radiation treatment for cervix cancer require a hemoglobin check at their first follow-up appointment.
- Patients who exhibit signs and symptoms of anemia at their first follow-up appointment require a hemoglobin check at their first follow-up appointment.
- Patients who completed their radiation treatment for cervix cancer require a hemoglobin check at their first follow-up appointment.
- After completing radiation treatment, ONLY patients who have symptomatic anemia, actively bleeding, or have a hemoglobin level less than 7 g/dL (4.34 mmol/L) require a blood transfusion.
- Comments

© 2021 Zayed S et al. *JAMA Network Open.*

SURVEY 3

Clinical Query: A patient will be undergoing curative intent radiation treatment for cervical cancer with External Beam Radiotherapy (EBRT) and brachytherapy.

Please consider the following definitions prior to proceeding:

- *Anemia for non-pregnant women: a hemoglobin concentration less than 12 g/dL which is equivalent to 120 g/L or 7.45 mmol/L.*
- *Threshold: The level at or below which an intervention would be indicated.*
- *Target: The goal level which was selected as the aim of an intervention.*

Please respond with your level of agreement to the following statements.

The Likert scale below will be used for every question:

Strongly Disagree	Disagree	Undecided	Agree	Strongly Agree

1, Strongly Disagree

2, Disagree

3, Undecided

4, Agree

5, Strongly Agree

EXTERNAL BEAM RADIOTHERAPY (EBRT)

EBRT: Timing of Hemoglobin Checks

- Hemoglobin levels should be checked weekly during treatment if the patient is receiving concurrent chemotherapy.
- Hemoglobin levels should be checked when clinically indicated: e.g. anemia upon presentation, signs or symptoms of anemia, active bleeding, post transfusion, and dropping hemoglobin levels.
- Comments

EBRT: Hemoglobin Transfusion Threshold

- For patients undergoing EBRT who are asymptomatically anemic, a hemoglobin level < 9 g/dL (5.59 mmol/L)* warrants a blood transfusion.
- Patients with a significant past medical history for cardiac disease (e.g. MI, CHF, etc.) should have a higher hemoglobin threshold for transfusion.
- Comments

EBRT: Hemoglobin Transfusion Target

- Patients who receive a blood transfusion should be transfused to a target hemoglobin of at least 9 g/dL (5.59 mmol/L)*.
- For patients with a significant past medical history for cardiac disease (e.g. MI, CHF, etc.) a higher transfusion target should be used.

© 2021 Zayed S et al. *JAMA Network Open.*

- Comments

EBRT: Transfusion Timing

- Patients should be transfused at any time (before, during, or after EBRT treatment) if they exhibit signs and/or symptoms of anemia.
- Patients should be transfused AFTER EBRT treatment if hemoglobin is below the pre-specified threshold for transfusion.
- Patients should be transfused AFTER EBRT treatment if clinically indicated e.g. signs and symptoms of anemia present, significant unanticipated bleeding during treatment, or hemoglobin level lower than 7 g/dL (4.34 mmol/L) etc.
- Comments

BRACHYTHERPAY

- Brachytherapy: Timing of Hemoglobin Checks
- Hemoglobin levels should be routinely checked within 24-48 hours BEFORE the start of every brachytherapy treatment.
- Hemoglobin levels should be checked DAILY if interstitial brachytherapy applicator is in situ for more than 24 hours.
- Hemoglobin levels should NOT be routinely checked AFTER every interstitial brachytherapy treatment.
- Comments/Justifications

Brachytherapy: Hemoglobin Transfusion Threshold

- For asymptomatically anemic patients undergoing interstitial or intracavitary brachytherapy, a hemoglobin level below 9 g/dL (5.59 mmol/L)* warrants a blood transfusion.
- Comments

Brachytherapy: Hemoglobin Transfusion Target

- Patients undergoing interstitial or intracavitary brachytherapy who receive a blood transfusion should be transfused to a target hemoglobin of at least 9 g/dL (5.59 mmol/L)*.
- A higher transfusion target should NOT be applied for patients undergoing interstitial brachytherapy with needle insertion compared to patients receiving intracavitary brachytherapy alone.
- For patients with a significant past medical history for cardiac disease (e.g. MI, CHF, etc.) a higher transfusion target should be used.
- Comments

Brachytherapy: Transfusion Timing

- Patients should be transfused if the hemoglobin level reaches the pre-specified transfusion threshold.
- Patients should be transfused at any time (before, during, or after brachytherapy treatment) if the patient is symptomatically anemic and/or their hemoglobin level is less than 7 g/dL (4.34 mmol/L).
- Comments

Follow-Up

- The FIRST follow-up appointment for patients who completed radiation treatment for cervix cancer should take place within 4-6 weeks after treatment completion, regardless of their transfusion requirements during treatment.
- Patients who completed their radiation treatment for cervix cancer do NOT routinely require a hemoglobin check at their first follow-up appointment.

© 2021 Zayed S et al. *JAMA Network Open.*

- All cervix cancer patients undergoing radiotherapy should be counselled on presenting to medical attention at their earliest convenience should they develop signs or symptoms of anemia, or if they are actively bleeding.
- Comments

© 2021 Zayed S et al. *JAMA Network Open.*
